# Clinic Characteristics and Antibiotic Prescribing for Acute Respiratory Infections in Japan

**DOI:** 10.1001/jamanetworkopen.2024.40406

**Published:** 2024-10-21

**Authors:** Ryuhei Aoyama, Yusuke Tsugawa, Masahiro Ishikane, Kei Kitajima, Daichi Sato, Atsushi Miyawaki

**Affiliations:** 1 Department of Health Services Research, Graduate School of Medicine, The University of Tokyo, Tokyo, Japan; 2 Faculty of Medicine, The University of Tokyo, Tokyo, Japan; 3 Division of General Internal Medicine and Health Services Research, David Geffen School of Medicine at UCLA, Los Angeles, California; 4 Department of Health Policy and Management, UCLA Fielding School of Public Health, Los Angeles, California; 5 Disease Control and Prevention Center, National Center for Global Health and Medicine, Tokyo, Japan; 6 M3, Inc, Akasaka Intercity, Tokyo, Japan; 7 Department of Clinical Epidemiology and Health Economics, School of Public Health, The University of Tokyo, Tokyo, Japan

## Abstract

This cross-sectional study examined clinic characteristics associated with antibiotic prescribing for acute respiratory infections in primary care in Japan.

## Introduction

Reducing inappropriate antibiotic prescribing for acute respiratory infections (ARIs) is crucial for combating antimicrobial resistance.^1^ Japan has implemented several outpatient antibiotic stewardship initiatives since 2016, with limited success.^2^ To enhance antibiotic stewardship, we examined clinic characteristics associated with antibiotic prescribing for ARIs in primary care in Japan.

## Methods

This cross-sectional study analyzed data from the Japan Medical Data Survey (JAMDAS), an electronic health record database of nationwide primary care clinics in Japan, compiled by M3, Inc.^3^ The University of Tokyo Ethics Committee approved this study and waived written informed consent because we used deidentified data. Details of data, measurements, and statistical analysis are provided in the eMethods in Supplement 1. Per the STROBE reporting guideline, we analyzed outpatient visits for nonbacterial ARIs (defined using the *International Statistical Classification of Diseases and Related Health Problems, Tenth Revision* codes of J00-J06 or J20-J22, excluding visits with codiagnoses for which antibiotics may be appropriate) for adults aged 18 to 99 years in clinics continuously observed in the JAMDAS database from October 1, 2022, to September 30, 2023. To focus on clinics engaged in adult primary care, we excluded clinics whose physician-owners had board certification in otolaryngology or pediatrics and clinics with fewer than 100 nonbacterial ARI visits in the study period. To examine the association of clinic characteristics with overall antibiotic prescribing, we used a multivariable logistic regression model that adjusted for visit characteristics and prefectures. Clinic characteristics included physician-owners’ sex and age (<45, 45-59, or ≥60 years), patient volume (clinic-level tertile of median number of daily patients), and group practice indicator (vs solo practice). To make national estimates, we used JAMDAS-provided clinic-level weights.^3^ Analyses were repeated with broad-spectrum antibiotic prescriptions (defined as third-generation cephalosporins, macrolides, and fluoroquinolones^1^) and the other antibiotic prescriptions as separate outcomes. To adjust for multiple comparisons, we used the Holm method and considered adjusted *P* < .05 as statistically significant.

## Results

After excluding 21 377 visits in 32 clinics with missing physician-owners’ age, we analyzed 977 590 ARI visits from 977 590 patients (mean [SD] age, 49.7 [20.1] years; 56.9% female) in 1183 clinics (Table 1). Antibiotics were prescribed for 171 483 visits (17.5%), with broad-spectrum antibiotics comprising 88.3% of the total antibiotic prescriptions. Clarithromycin was most commonly prescribed (52 662 visits [30.7%]), followed by levofloxacin (20 948 visits [12.2%]), cefditoren (19 260 visits [11.2%]), azithromycin (19 080 visits [11.1%]), cefcapene (15 802 visits [9.2%]), and amoxicillin (13 591 visits [7.9%]). Antibiotics were more likely to be prescribed in clinics owned by older physicians (adjusted odds ratio [AOR] for ≥60 vs <45 years, 2.14; 95% CI, 1.56-2.92; adjusted *P* < .001) and clinics with higher patient volumes (AOR for high vs low, 1.47; 95% CI, 1.11-1.96; adjusted *P* = .02) (Table 2). Group practices were less likely to prescribe antibiotics than solo practices (AOR, 0.71; 95% CI, 0.56-0.89; adjusted *P* = .01). We found no evidence that antibiotic prescription proportions differed by physician-owner’s sex. Similar associations were found for broad-spectrum antibiotics but not for the other antibiotics.

**Table 1.** Characteristics of Patients With Acute Respiratory Infections From October 2022 to September 2023 and Characteristics of Physicians and Visits Where They Received Care (National Estimates From the Japan Medical Data Survey)

Characteristic	Weighted No. (%)^a^
No. of clinics	1183
Physician-owner sex	
Female	97 (8.2)
Male	1086 (91.8)
Physician-owner’s age, mean (SD), y	62.8 (11.8)
Physician-owner’s age category	
≤44 y	93 (7.8)
45-59 y	342 (28.9)
≥60 y	748 (63.3)
Patient volume^b^	
Low	380 (32.1)
Medium	388 (32.8)
High	415 (35.1)
Solo vs group practice	
Solo	628 (53.1)
Group	555 (46.9)
No. of visits	977 590
Sex	
Female	555 882 (56.9)
Male	421 708 (43.1)
Age, mean (SD), y	49.7 (20.1)
Age category, y	
18-39	362 316 (37.1)
40-59	302 443 (30.9)
60-79	217 842 (22.3)
80-99	94 990 (9.7)
Charlson Comorbidity Index	
0	689 059 (70.5)
1	165 501 (16.9)
≥2	123 030 (12.6)
Place of care	
In person	968 844 (99.1)
Telemedicine	8746 (0.9)

^a^ To provide national estimates, we used clinic-level weights; therefore, numbers in each category may not sum exactly to the total number.

^b^ We categorized patient volume as low (≤35 visits per day), medium (36-57 visits per day), or high (≥58 visits per day) based on the clinic-level tertile of the median number of patients seen per day from October 1, 2022, to September 30, 2023.

**Table 2.** Associations Between Clinic Characteristics and Antibiotic Prescriptions for Adults With Acute Respiratory Infections^a^

Characteristic	Weighted No. of visits	For overall antibiotics				For prescribing broad-spectrum antibiotics^b^				For prescribing other antibiotics^b^			
		% (95% CI)	AOR (95% CI)	Unadj *P* value	Adj *P* value^c^	% (95% CI)	AOR (95% CI)	Unadj *P* value	Adj *P* value^c^	% (95% CI)	AOR (95% CI)	Unadj *P* value	Adj *P* value^c^
Physician-owner’s sex													
Male	913 061	17.1 (15.7-18.6)	1 [Reference]	NA	NA	15.1 (13.8-16.5)	1 [Reference]	NA	NA	2.0 (1.6-2.4)	1 [Reference]	NA	NA
Female	64 529	23.5 (16.6-30.3)	1.52 (1.00-2.32)	.05	.10	20.8 (15.1-26.4)	1.50 (1.02-2.21)	.04	.07	2.7 (1.2-4.1)	1.34 (0.72-2.49)	.35	>.99
Physician-owner’s age, y													
<45	111 703	10.2 (7.8-12.5)	1 [Reference]	NA	NA	8.3 (6.0-10.5)	1 [Reference]	NA	NA	1.9 (1.4-2.4)	1 [Reference]	NA	NA
45-59	288 594	18.0 (15.3-20.6)	2.00 (1.42-2.80)	<.001	<.001	16.0 (13.5-18.5)	2.18 (1.51-3.14)	<.001	<.001	2.0 (1.5-2.5)	1.05 (0.70-1.58)	.81	>.99
≥60	577 293	18.9 (16.8-21.0)	2.14 (1.56-2.92)	<.001	<.001	16.8 (14.8-18.7)	2.31 (1.65-3.25)	<.001	<.001	2.2 (1.6-2.7)	1.14 (0.77-1.68)	.52	>.99
Patient volume													
Low	169 759	14.8 (12.0-17.6)	1 [Reference]	NA	NA	13.0 (10.5-15.6)	1 [Reference]	NA	NA	1.8 (1.2-2.3)	1 [Reference]	NA	NA
Medium	282 902	15.0 (12.9-17.1)	1.01 (0.76-1.35)	.92	.92	13.4 (11.4-15.3)	1.03 (0.77-1.38)	.85	.85	1.6 (1.2-2.1)	0.91 (0.59-1.42)	.68	>.99
High	524 929	20.0 (17.7-22.3)	1.47 (1.11-1.96)	.008	.02	17.6 (15.5-19.7)	1.45 (1.09-1.95)	.01	.04	2.4 (1.8-3.1)	1.38 (0.91-2.10)	.13	.80
Solo vs group practice													
Solo	466 635	20.2 (17.7-22.6)	1 [Reference]	NA	NA	18.1 (15.8-20.3)	1 [Reference]	NA	NA	2.1 (1.6-2.7)	1 [Reference]	NA	NA
Group	510 955	15.4 (13.6-17.2)	0.71 (0.56-0.89)	.003	.01	13.4 (11.7-15.0)	0.69 (0.55-0.86)	.001	.004	2.0 (1.5-2.5)	0.95 (0.66-1.38)	.80	>.99

Abbreviatons: Adj, Adjusted; AOR, adjusted odds ratio; NA, not applicable; Unadj, Unadjusted.

^a^ We analyzed visits for acute respiratory tract infections that occurred in 1183 primary care physicians’ clinics in the Japan Medical Data Survey database between October 2022 and September 2023. We examined the associations of clinic characteristics and antibiotic prescribing using a logistic regression model that adjusted for visit characteristics (patients’ sex, age, and Charlson Comorbidity Index score, indicators of months, indicators of dates, and an indicator of telemedicine visits) and prefectures where clinics were located. Clinic-level weights were applied. Standard errors were clustered at the clinic level. Adjusted proportions were calculated using predictive margins.

^b^ Broad-spectrum antibiotics consisted of third-generation cephalosporins, macrolides, and fluoroquinolones.

^c^ To account for multiple comparisons (6 comparisons), we adjusted *P* values using the Holm method. We considered adjusted *P* < .05 statistically significant.

## Discussion

Broad-spectrum antibiotics comprised most antibiotics for ARIs. Promotion of the appropriate use of broad-spectrum antibiotics is needed.^4^ High patient volume clinics may overprescribe antibiotics because of time pressure or decision fatigue. This is important as Japanese physicians see twice as many outpatients as the Organisation for Economic Cooperation and Development, on average.^5^ Group practices may reduce antibiotic prescribing because of lower individual workload and more peer discussions.^6^ Older physicians may need updated antibiotic use training because of limited prior opportunities. Limitations included relying on administrative diagnosis codes, lack of comparison before and after the pandemic onset, and limited generalizability to clinics outside JAMDAS or to other clinical settings. In this cross-sectional study of nationwide primary care clinics in Japan, higher-volume clinics, solo practices, and clinics owned by older physicians were more likely to prescribe antibiotics, especially broad-spectrum antibiotics, for nonbacterial ARIs. Our findings could help policymakers implement more targeted interventions in ongoing antibiotic stewardship efforts.

## Notes

Supplement 1.
eMethods.

eReferences.

Supplement 2.
Data Sharing Statement
