# Supplementary material for: Clinic Characteristics and Antibiotic Prescribing for Acute Respiratory Infections in Japan
Source: JAMA Netw Open. 2024 Oct 21;7(10):e2440406. doi: 10.1001/jamanetworkopen.2024.40406 (PMC11581480; doi:10.1001/jamanetworkopen.2024.40406)
Supplement: Supplement 1. — eMethods. eReferences. [file jamanetwopen-e2440406-s001.pdf]

Supplemental Online Content

Aoyama R, Tsugawa Y, Ishikane M, Kitajima K, Sato D, Miyawaki A. Clinic characteristics and antibiotic prescribing for acute respiratory infections in Japan. *JAMA Netw Open*. 2024;7(10):e2440406. doi:10.1001/jamanetworkopen.2024.40406

eMethods.
eReferences.

This supplemental material has been provided by the authors to give readers additional information about their work.

© 2024 Aoyama R et al. *JAMA Network Open.*

eMethods.

Data

This cross-sectional study analyzed data from the Japan Medical Data Survey (JAMDAS), collected and compiled by M3, Inc. (Tokyo, Japan).[1] JAMDAS collects electronic health record (EHR) data linked with claims data from clinics across Japan, including all the data on outpatient visits such as in-person (office, home, or nursing home) visits and telemedicine (telephone or video) visits. JAMDAS includes patient-level information on patients' diagnoses, prescriptions, and medical practices provided and has been used in several studies.[2,3] The number of visits to clinics continuously enrolled in JAMDAS from October 2022 to September 2023 was approximately 40 million, accounting for approximately 4% of all primary care visits in Japan.[4] Compared to nationally representative estimates of primary care visits from the Patient Survey (a government statistical survey),[5] patients registered in the JAMDAS have a similar sex composition to those in the Patient Survey and tend to be slightly younger (In JAMDAS vs. patient survey, the percentages of female patients were 58.8% vs. 58.3%, respectively. By age group, 15.5% vs. 14.0% for those under age 20, 42.2% vs. 29.4% for those aged 20–59, and 42.3% vs. 56.7% for those aged 60 and over, respectively.). The JAMDAS also contains clinic information, including owner-physicians' sex and age and the prefectures where clinics are located.

Patient Population

We analyzed outpatient visits due to non-bacterial acute respiratory infections (ARIs) for adults aged 18 to 99 years in clinics continuously observed in the JAMDAS database during the study period from October 1, 2022, to September 30, 2023. This period was chosen because mobility and healthcare utilization restrictions for the COVID-19 pandemic were not implemented in Japan after October 2022, and the direct impact of public health interventions on healthcare utilization patterns, such as antibiotic prescription, was considered minimal. ARIs were defined using the *International Classification of Diseases, Tenth Edition* (ICD-10) code of J00–J06 or J20–J22, following previous studies conducted in Japan.[6,7] We analyzed visits for which the date of ARI diagnosis corresponded with the date of the visit (i.e., excluding revisits with the same diagnosis after the initial visit).[2] To identify non-bacterial ARIs, we excluded visits with co-diagnoses for which antibiotics may be appropriate, comprising acute sinusitis (J01), acute

© 2024 Aoyama R et al. *JAMA Network Open.*

pharyngitis (J02), tonsillitis (J03), acute tracheitis (J04.1), acute epiglottitis (J05.1), other acute upper respiratory infections of multiple sites (J06.8), unspecified acute lower respiratory infection (J22), acute bacterial pneumonia (ICD-10 code J13–J18), otitis media (H66), peritonsillar abscess (J36), and chronic pharyngitis (J31.2) (47.1% of the 2,331,825 ARI visits).[8] Furthermore, to focus on clinics primarily engaged in adult primary care, including the care of ARIs, we excluded patients treated in clinics whose physician-owners had board certification in otolaryngology or pediatrics or clinics with <100 non-bacterial ARI visits in the study period (19.1% of the remaining 1,234,404 visits). We also excluded patients treated in clinics whose physician owners' age was missing (2.1% of the remaining 998,967 visits). The distribution of sex and age of patient visits was similar between clinics without and with missing physician-owners' age (unweighted values, 56.2% vs. 57.3% female, mean age 47.4 vs. 46.8 years).

Clinic Characteristics

The clinic characteristics of interest were the physician-owners' sex (male or female) and age (<45, 45–59, or ≥60 years), patient volume, and an indicator of group practice (vs. solo practice). We categorized patient volume as low (≤ 35 visits per day), medium (36–57 visits per day), or high (≥ 58 visits per day) based on the clinic-level tertile of the median number of patients seen per day from October 1, 2022, to September 30, 2023. A group practice was defined as a practice with multiple physicians working in the same clinic.

Outcomes

The primary outcome was the prescription of an oral antibiotic agent. The antibiotics were classified according to the anatomical therapeutic chemical classification system developed by the World Health Organization Collaborating Centre for Drug Statistics Methodology.[9] We defined the antibiotic agents as "J01" (anti-infectives for systemic use). The secondary outcome was the use of broad-spectrum antibiotics and the use of other antibiotics. Recognizing the differences in the definition of broad-spectrum antibiotics, we categorized broad-spectrum antibiotics as third-generation cephalosporins, macrolides, and fluoroquinolones. This was because the majority of broad-spectrum antibiotics used for ARIs in Japan are one of these 3 classes[7] and their use is the principal target of measures in Japan's National Action Plan on AMR (reference 1 in the main manuscript).

Adjustment Variables

We adjusted for visit characteristics and indicators of prefectures where the clinics were located. The visit characteristics were patients' sex, age (in increments of 5 years except for 18–19 years), and Charlson Comorbidity Index (CCI) score (categorized as 0, 1, or ≥2),[10] indicators of months of visits, indicators of dates, and an indicator of telemedicine visits (vs. in-person visits) We defined comorbidities included in the CCI according to the ICD-10 diagnosis codes assigned to patients at the time of the first visit during the study period.

Statistical Analysis

First, we described the characteristics of clinics and ARI visits. Second, we described the breakdown of antibiotic prescriptions for patients with ARIs.

Third, we examined the associations of clinic characteristics with antibiotic prescribing, using a multivariable logistic regression model that adjusted for patient characteristics and prefectures (see **Adjustment Variables**). We used standard errors clustered at the clinic level to account for within-clinic correlation. We also calculated the risk-adjusted antibiotic prescription proportions for each clinic category, using the marginal standardization form of predictive margins.[11] We repeated the analyses by setting broad-spectrum antibiotic prescriptions and the other antibiotic prescriptions separately as outcomes.

To adjust for multiple comparisons (6 comparisons), we used the Holm method,[12] which sequentially compares the ith smallest P-value (for $i = 1, ..., 6$) among the 6 original P-values with progressively less restrictive alpha levels ($=0.05/(6 - i+1)$). To make the interpretation easier, we calculated the adjusted P-value by multiplying the unadjusted P-values by ($6 - i+1$) times and considered an adjusted P-value of <0.05 to be statistically significant. All analyses were conducted using Stata version 17 (StataCorp LLC).

Weighing Method

The JAMDAS database is not a random sample of primary care clinics in Japan. Since the JAMDAS consists of clinics that have introduced the M3 EHR system, they may not represent primary care clinics nationwide because clinics that introduce the EHR system might have different characteristics from those that do not. To extrapolate the

© 2024 Aoyama R et al. *JAMA Network Open.*

JAMDAS sample primary care clinics to all the primary care clinics in Japan and make national estimates, the JAMDAS provides clinic-level weights. These JAMDAS-provided weights were developed by calculating the inverse of the estimated probability of inclusion in the JAMDAS among all Japanese primary care clinics (inclusion probability) as conducted in prior studies.[2,3]

The inverse probability was derived using a nationwide medical facility database. This nationwide medical facility database (called the Doctor Computer File [DCF] database) is a database of medical institutions throughout Japan collected by Nihon Ultmark Inc. (a subsidiary company of M3 Inc.) for business purposes and has been used in academic research.[13,14] This database includes information on all medical facilities (including primary care clinics) in Japan and physicians working at medical facilities (sex, age, titles, and specialties) based on publicly available official statistics and information collected by medical representatives of pharmaceutical companies. Primary care clinics were matched from the two databases (clinics in the JAMDAS and nationwide medical facility databases), using facility identifiers. A multivariable logistic regression model was conducted to estimate the inclusion probability of clinics in the JAMDAS database among all Japanese primary care clinics. The explanatory variables in the logistic model included factors that may influence the introduction of an EHR system, including prefecture fixed effects; region; number of beds (in Japan, a small number of primary care clinics are allowed by law to have a few [<19] beds); number of physicians in the clinic; clinic owner's sex, age, and specialties (if any other than primary care physician status); and quadratic terms of all of these variables. The final JAMDAS-provided weight was standardized to keep the total number of patients constant.

eReferences.

1. About M3 Inc. M3 Inc. Accessed March 18, 2024. https://corporate.m3.com/en/corporate/

2. Miyawaki A, Kitajima K, Iwata A, Sato D, Tsugawa Y. Antibiotic prescription for outpatients with COVID-19 in primary care settings in Japan. *JAMA Netw Open*. 2023;6(7):e2325212. doi:10.1001/jamanetworkopen.2023.25212

3. Miyawaki A, Kitajima K, Iwata A, Sato D, Tsugawa Y. Physician characteristics associated with antiviral prescriptions for older adults with COVID-19 in Japan: an observational study. *BMJ Open*. 2024;14(3):e083342. doi:10.1136/bmjopen-2023-083342

4. Ministry of Health Labour and Welfare Japan. Statistics of Medical Care Activities in Public Health Insurance. Published June 15, 2024. Accessed June 15, 2024. https://www.mhlw.go.jp/toukei/list/26-19.html

5. Ministry of Health Labour and Welfare. Patient Survey 2020. Accessed June 16, 2024. https://www.mhlw.go.jp/toukei/saikin/hw/kanja/20/index.html

© 2024 Aoyama R et al. *JAMA Network Open.*

6. Kimura Y, Fukuda H, Hayakawa K, et al. Longitudinal trends of and factors associated with inappropriate antibiotic prescribing for non-bacterial acute respiratory tract infection in Japan: A retrospective claims database study, 2012–2017. *PloS one*. 2019;14(10):e0223835. doi:10.1371/journal.pone.0223835

7. Tsuzuki S, Kimura Y, Ishikane M, Kusama Y, Ohmagari N. Cost of inappropriate antimicrobial use for upper respiratory infection in Japan. *BMC Health Serv Res*. 2020;20(1):153. doi:10.1186/s12913-020-5021-1

8. Chua KP, Fischer MA, Linder JA. Appropriateness of outpatient antibiotic prescribing among privately insured US patients: ICD-10-CM based cross sectional study. *BMJ*. 2019;364(8183):k5092. doi:10.1136/bmj.k5092

9. World Health Organization. The ATC/DDD Methodology. Accessed June 19, 2024. https://www.who.int/tools/atc-ddd-toolkit/methodology

10. Quan H, Li B, Couris CM, et al. Updating and validating the charlson comorbidity index and score for risk adjustment in hospital discharge abstracts using data from 6 countries. *Am J Epidemiol*. 2011;173(6):676-682. doi:10.1093/aje/kwq433

11. Williams R. Using the margins command to estimate and interpret adjusted predictions and marginal effects. *SJ*. 2012;12(2):308-331. doi:10.1177/1536867X1201200209

12. Holm S. A simple sequentially rejective multiple test procedure. *Scand J Stat*. 1979;6(2):65-70. Accessed November 30, 2020. http://www.jstor.org/stable/4615733

13. Kamitani S, Nakamura F, Itoh M, Sugiyama T, Toyokawa S, Kobayashi Y. Differences in medical schools' regional retention of physicians by school type and year of establishment: effect of new schools built under government policy. *BMC Health Serv Res*. 2015;15:581. doi:10.1186/s12913-015-1240-2

14. Takaku R. How is increased selectivity of medical school admissions associated with physicians' career choice? A Japanese experience. *Human Resources for Health*. 2020;18(1):38. doi:10.1186/s12960-020-00480-0

© 2024 Aoyama R et al. *JAMA Network Open.*
