# Supplementary material for: Clinic Characteristics and Antibiotic Prescribing for Acute Respiratory Infections in Japan
Source: JAMA Netw Open. 2024 Oct 21;7(10):e2440406. doi: 10.1001/jamanetworkopen.2024.40406 (PMC11581480; doi:10.1001/jamanetworkopen.2024.40406)
Supplement: Supplement 2. — Data Sharing Statement [file jamanetwopen-e2440406-s002.pdf]

Data Sharing Statement

Aoyama. Clinic Characteristics and Antibiotic Prescribing for Acute Respiratory Infections in Japan. *JAMA Netw Open*. Published October 21, 2024.
doi:10.1001/jamanetworkopen.2024.40406

Data
Data available: No

Additional Information
Explanation for why data not available: The JAMDAS database is proprietary data owned by M3, Inc., and therefore, cannot be shared. Those individuals who are interested in using the JAMDAS data should contact Yusuke Tsugawa (ytsugawa@mednet.ucla.edu).
